# Toward fairness in artificial intelligence for medical image analysis: identification and mitigation of potential biases in the roadmap from data collection to model deployment

**DOI:** 10.1117/1.JMI.10.6.061104

**Published:** 2023-04-26

**Authors:** Karen Drukker, Weijie Chen, Judy Gichoya, Nicholas Gruszauskas, Jayashree Kalpathy-Cramer, Sanmi Koyejo, Kyle Myers, Rui C. Sá, Berkman Sahiner, Heather Whitney, Zi Zhang, Maryellen Giger

**Affiliations:** a The University of Chicago, Department of Radiology, Chicago, Illinois, United States; b US Food and Drug Administration, Division of Imaging, Diagnostics, and Software Reliability, Office of Science and Engineering Laboratories, Center for Devices and Radiological Health, Silver Spring, Maryland, United States; c Emory University, Department of Radiology, Atlanta, Georgia, United States; d University of Colorado Anschutz, Department of Ophthalmology, Aurora, Colorado, United States; e Stanford University, Department of Computer Science, Stanford, California, United States; f Puente Solutions LLC, Phoenix, Arizona, United States; g National Institutes of Health, Bethesda, Maryland, United States; h University of California, San Diego, La Jolla, California, United States; i Jefferson Health, Philadelphia, Pennsylvania, United States

**Keywords:** artificial intelligence, machine learning, bias, fairness

## Abstract

**Purpose:**

To recognize and address various sources of bias essential for algorithmic fairness and trustworthiness and to contribute to a just and equitable deployment of AI in medical imaging, there is an increasing interest in developing medical imaging-based machine learning methods, also known as medical imaging artificial intelligence (AI), for the detection, diagnosis, prognosis, and risk assessment of disease with the goal of clinical implementation. These tools are intended to help improve traditional human decision-making in medical imaging. However, biases introduced in the steps toward clinical deployment may impede their intended function, potentially exacerbating inequities. Specifically, medical imaging AI can propagate or amplify biases introduced in the many steps from model inception to deployment, resulting in a systematic difference in the treatment of different groups.

**Approach:**

Our multi-institutional team included medical physicists, medical imaging artificial intelligence/machine learning (AI/ML) researchers, experts in AI/ML bias, statisticians, physicians, and scientists from regulatory bodies. We identified sources of bias in AI/ML, mitigation strategies for these biases, and developed recommendations for best practices in medical imaging AI/ML development.

**Results:**

Five main steps along the roadmap of medical imaging AI/ML were identified: (1) data collection, (2) data preparation and annotation, (3) model development, (4) model evaluation, and (5) model deployment. Within these steps, or bias categories, we identified 29 sources of potential bias, many of which can impact multiple steps, as well as mitigation strategies.

**Conclusions:**

Our findings provide a valuable resource to researchers, clinicians, and the public at large.

## 1 Introduction

Artificial intelligence and machine learning (AI/ML) in medical imaging provide important methods for leveraging large amounts of data to build models to detect disease and provide diagnosis, prognosis, and risk assessment tools to support decision-making in medicine. The development and deployment of AI/ML models in a wide variety of fields have grown substantially in the past decade, broadening considerations for more careful evaluation of the algorithms. Researchers and developers note that there are persistent challenges in generalizability and bias along the model development and deployment pipeline, including the characteristics of data used in training models and testing them. These issues have been noted and studied in fields, such as economics and sociology^1^ as well as the natural sciences.^2^ Efforts to understand and address bias in the broader AI/ML literature involve expanding fairness metrics and definitions to include additional factors^3^ and the development of mitigation strategies. These concerns are especially important in healthcare and medicine broadly^4^ and have been an area of study in medical imaging by several groups and initiatives, including studies which demonstrate the potential for bias in medical imaging of COVID-19.^5^ Recently, a three-part series^6^^–^^8^ published in *Radiology: Artificial Intelligence* presented methods and insights for mitigating bias in AI/ML for medical imaging through considerations for data handling,^6^ model development,^7^ and performance metrics.^8^

The capabilities of AI/ML in medical imaging are especially relevant for addressing the COVID-19 crisis, for which there exist crucial needs in distinguishing COVID-19-related disease from other similar clinical presentations, finding incidental disease, and developing medical imaging protocols and tools for addressing long COVID-19. The Medical Imaging Data and Resource Center (MIDRC^9^) is a multi-institutional effort supported by the National Institute of Biomedical Imaging and Bioengineering of the National Institutes of Health, which aims to develop a carefully curated imaging data commons and to gather and produce resources to foster and accelerate clinical translation of AI/ML models, with fairness, trust, and equity as guiding principles. MIDRC seeks to (1) provide an open, unbiased, representative health data resource for all, lowering the possibility of statistical fallacies and representational errors and (2) to develop and share tools, machine learning algorithms, and analytical methods for discovery, visualization and understanding of diverse data sets. MIDRC is committed to the principles of equity and inclusion and actively promotes diversity within the MIDRC leadership and research teams, as noted in Ref. 10. A diverse data collection and curation strategy, as well as the mitigation of bias in data analysis within the MIDRC commons, are crucial to yield ethical AI algorithms that produce trustworthy results for all groups. MIDRC aims to collect imaging data that are representative of the population and actively seeks data contributions from rural and underrepresented community hospitals and smaller healthcare systems. It has been collecting a large, diverse, and representative open data set for the development of fair and equitable medical imaging AI/ML models to help combat the ongoing and evolving COVID-19 pandemic. To date, more than 150,000 imaging studies (mainly chest radiographs and CTs) and associated metadata have been collected and are being curated and harmonized, with more than 100,000 imaging studies currently freely downloadable from the MIDRC user portal hosted on the Gen3 data commons platform.^11^ The diversity of the data is tracked, and tools are shared for understanding performance metrics and various biases, allowing all investigators to access data and resources.

**Fig. 1:**
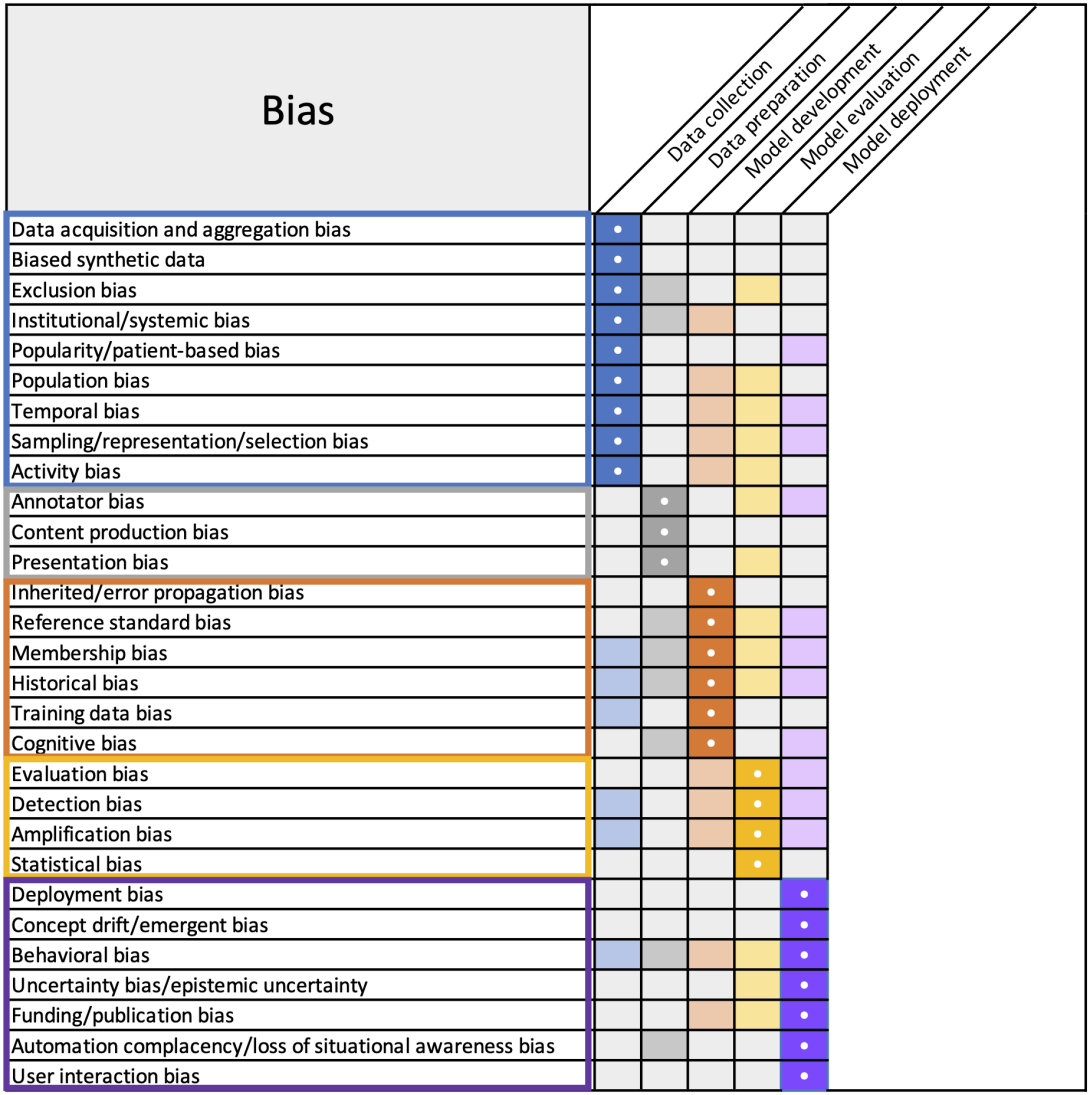
Overview of potential biases and where they are most likely to occur along the medical imaging AI/ML pipeline. The dark shading with white dot indicates the most likely occurrence and lighter shading indicates additional potential occurrences.

**Fig. 2:**
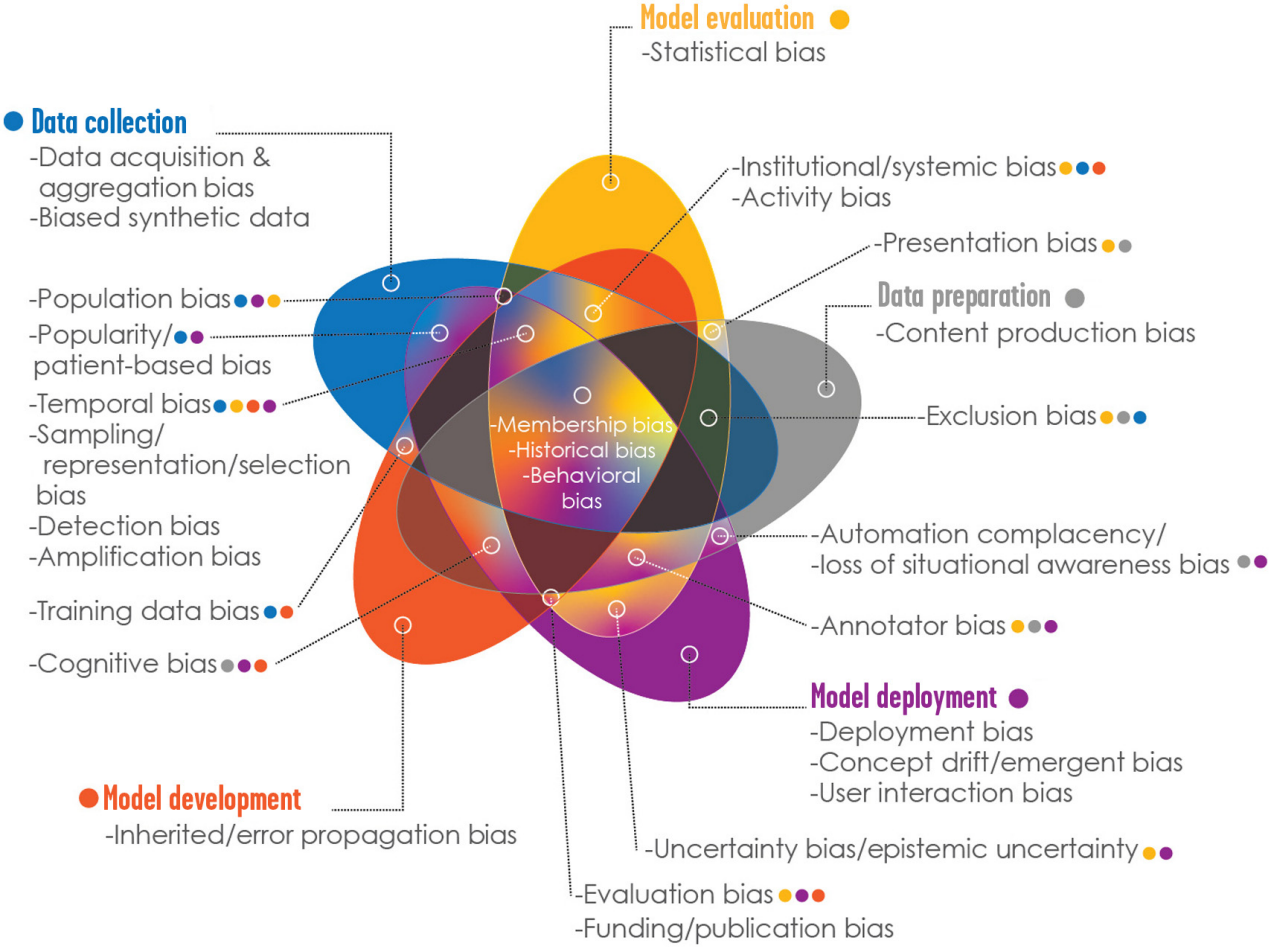
Venn diagram of the identified potential biases and main bias categories (Fig. 1). Biases associated with a single category are listed under the applicable category. For biases associated with multiple categories, colored dots indicate which category each bias belongs to.

The bias “library” provided in this manuscript is a foundational contribution by the MIDRC Bias and Diversity Working Group, to support investigators in their use of the MIDRC data and medical imaging data in general. The purpose is to share resources for the identification, characterization, and mitigation of bias in AI/ML along steps in the pipeline for AI/ML in medical imaging. The types of biases coalesced around five main areas: (1) data collection, (2) data preparation and annotation, (3) model development, (4) model evaluation, and (5) model deployment (Figs. 1 and 2). The biases studied and presented here were identified within the context of COVID-19 imaging as a part of the aims of MIDRC and are relevant for medical imaging broadly. For each bias identified, we reviewed the literature and collected available information for the quantification and mitigation of biases. This resource provides a comprehensive overview of bias that will serve researchers, clinicians, and the public at large in their efforts to identify, measure, and mitigate bias in AI/ML in medical imaging.

## 2 Bias Categories and Potential Biases

Figure 1 provides a summary of the identified potential biases along the medical imaging AI/ML pipeline and the main bias categories, or stages, coding the potential sources of bias by color and indicating with dark shades the most likely stage at which a given bias might arise. The lighter shades along each row make clear that a given source of bias might enter the process at more than one stage. Another representation is provided in Fig. 2, which emphasizes the overlaps in membership of bias categories for each potential bias.

## 3 Bias Category I: Data Collection

Nine sources of bias mainly related to data collection were identified (Table 1) and are discussed below.

**Table 1:** Overview of biases mainly related to data collection.

Bias source	Definition	Potential mitigation
Data acquisition and generation bias	Is introduced when data (i) comes from limited acquisition sources, (ii) was collected under different standard processes, or (iii) was duplicated due to repeat collection or acquisition	For (i) and (ii), either collect enough cases from all acquisition types or train specifically for an acquisition type and use “only” for that acquisition type. For (iii), use GUIDs (globally unique identifiers) so that the data source can be traced to avoid duplicate use
Biased synthetic data	Arises from the addition of biased synthetic data to a dataset	Carefully consider (e.g., with a list of known potential biases) the kinds of biases synthetic data generation may be adding to a small or already-biased data set
Exclusion bias	Is introduced (i) when specific population groups are excluded from data collection, training, testing or subsequent analyses, or (ii) when some features from the dataset are excluded in AI/ML model training	(i) Ensure that the training sample is representative of the population the AI/ML model is intended for and carefully examine inclusion and exclusion criteria. (ii) Perform sufficient analysis before discarding features from the training dataset
Institutional/systemic bias	Institutional/systemic bias occurs when the procedures and practices of institutions result in certain social groups being advantaged or favored and others being disadvantaged, devalued, or treated differently	Carefully review patient management at all institutions that provide data to minimize institutional bias
Popularity/patient-based bias	Occurs when current trends influence patients’ decision-making whether to undergo a specific test, which subsequently affects data collection	Be aware of trends in imaging recommendations and patient adherence to those recommendations
Population bias	Arises when statistics, demographics, and characteristics differ between the original target population and the population represented in the actual dataset or platform	Data harmonization procedures to account for differences in populations
Temporal bias	Arises from (i) differences in populations and behaviors over time, (ii) the use of data that is not representative of diagnostic clinical data, or (iii) the correlation of clinician/reader performance and state of knowledge of the disease	Define clear baseline dates in cohort studies. Regular postdeployment performance assessment may indicate the need for intervention
Sampling/representation/selection bias	Occurs when patient data used for training/tuning/testing an AI/ML model is not representative of the patient population to which the algorithm is intended to be applied	Data collection from multiple sources; curation by matching key characteristics of the intended patient population
Activity bias	Occurs when models are trained with data from regions or clinical sites that are active in using certain modalities (e.g., imaging specialties), archiving data, and developing models	Emphasize effort in collecting data from less active regions. Model retraining or recalibration in deployment

### 3.1 Data Acquisition and Aggregation Bias

Imaging data are often collected from one acquisition source (single hospital, clinic, and imaging provider) or a limited number of image acquisition sources, thus not covering a representative range of image acquisition modalities, manufacturers, scanner models, or protocols, resulting in data generation bias. If AI/ML models are trained or tested with only one or a limited number of acquisition types, they may not generalize to all acquisition types. Data acquisition and aggregation bias^4^ can occur, e.g., when the training data for an AI/ML model comes from different CT scanner manufacturers than those that will be used in testing the algorithm (related to training data bias). It can also occur when data from a single or a few CT scanner manufacturers are used in training and in testing of an AI/ML model, a situation that is not representative of the real-world conditions the algorithm will encounter once deployed (related to deployment bias). As the name implies, this type of bias is focused on to how data are generated, and it is not limited to real patient data. Many contemporary AI/ML models use synthetic data, and if the synthetic data are generated in a way that limits its representation of the intended data acquisition devices, then its use will also result in data acquisition bias. Data generation bias can lead to equity concerns when there are systematic correlations between devices and patient demographics, e.g., systematic differences in medical imaging acquisition devices and modalities across continents, especially compared to the global south. Other related types of biases, such as population bias (focused on the characteristics of the population in the training, test, and clinical use situations) and training data bias (inclusive of data generation bias, but with a broader range of characteristics), are discussed in different sections below.

Another potential cause of data acquisition and aggregation bias is the use of multiple public datasets (or a single public set aggregated from multiple datasets), which may result in inclusion of partially the same data labeled with different identifiers (resulting in so-called Frankenstein datasets) so that training and test sets may unknowingly not be independent. Mitigation strategies for data acquisition and aggregation bias include the collection of enough cases from all relevant acquisition types and, for public datasets, the use of GUIDs (globally unique identifiers) so that the data source can be traced and duplicate use of the same case(s) with different identifiers can be avoided. Datasheets^12^ can also be used for transparency of the data sources, and to clarify use-cases that might require more care.

### 3.2 Biased Synthetic Data

Synthetic data are often offered as a mitigation technique to reduce bias that may be present in real datasets^13^ and have been successfully used for this purpose.^14^^,^^15^ Carefully crafted synthetic data can indeed be used to mitigate bias, but one should not assume that synthetic data generation will automatically remedy biased data collection and data bias. Since many types of synthetic data are generated using a procedure or algorithm that itself needs to be trained, the biases present in a real data set may creep into the synthetic data. For example, if the synthetic data generation is based on too few samples, and those samples all contain a particular type of bias, then the bias will be incorporated into the generated synthetic data. In addition, the procedures used for generating synthetic data, both for methods that need to be trained and for models that are based on knowledge or physics, may introduce additional bias. For example, for a physics-based model, inaccuracies in the data generation pipeline could lead to a bias. Biased synthetic data, when it contains demographic biases, can exacerbate downstream equity concerns. Careful study, design,^16^ and testing are needed to determine if synthetic data are helpful in mitigating bias for each individual task and does not introduce new biases.

### 3.3 Exclusion Bias

Population groups may be systematically excluded and hence underrepresented in the training of an AI/ML model, which results in exclusion bias.^17^ This exclusion effect is even more profound when patients with a particular condition are excluded differently for different types of outcomes.^18^ For example, this can occur when patients negative for the condition of interest with a certain co-morbidity are *excluded* but patients positive for this condition with the same co-morbidity are included in the sample. Another example of exclusion bias might be the exclusion of portable chest radiographs from the data set for a certain task. Since patients imaged with portable radiography are likely to be sicker, this may result in distortion of the patient population with more severe disease. Consequently, the AI/ML model may not be able to perform accurately in a subset of a population that is excluded from the data collection, resulting in a biased model if exclusion bias happens in the training data, and/or in biased performance estimates if exclusion bias happens in the test data. Thus if inclusion and exclusion criteria are not carefully described and justified, severe bias may occur both in model training and testing. Exclusion bias may also arise in data preparation and annotation as well as model evaluation. Exclusion bias may be mitigated by better data sampling and clear data description, including possible limitations.

### 3.4 Institutional/Systemic Bias

There may be established practices in an institution that result in different social groups being managed differently.^19^ These practices may sometimes not be conspicuous unless a special effort is made to highlight them. This need not be the result of any conscious prejudice or discrimination but rather stemming from simply following existing rules or norms. This bias can be introduced in data collection and in model development, especially when the reference standard is defined by how cases are managed by the institution. For example, consider a situation in which data are being collected to develop a prediction model for patient admission to an ICU. The model is trained and validated with the reference standard defined as “patient admitted/not admitted to ICU.” If there is a bias in admitting patients to the ICU, this bias will taint the reference standard and then be propagated to the developed model. Thus one needs to carefully review patient management at all institutions that provide data to minimize institutional bias.

### 3.5 Popularity/Patient-Based Bias

Popularity bias is often noted with AI/ML systems that recommend products, movies, books, and other media based upon their popularity with similar users.^20^ Humans tend to make decisions based not only on facts but also based on perceptions and influence. Popularity bias may cause changes in the data collected based on the trends of the day. Such could happen in medical imaging AI/ML if the available data or selection of models is influenced by trends rather than careful consideration of scientific evidence. If findings and statistics are skewed based on current trends, long-term analyses may give biased findings and require temporal correction. For example, the decision for women to undergo screening mammography has been known to be influenced by positive or negative articles in the popular press. When different population groups are influenced differently by the press or trends, the bias in available data will result in an especially disparate impact of AI/ML models.

### 3.6 Population Bias

Population bias occurs when the characteristics of a training data population are different from the characteristics of a testing data population.^21^ The characteristics may include biological differences, demographic differences, social differences (such as the impacts of socioeconomic differences on access to health care) or technical differences in image acquisition that correlate to demographic differences. Population bias is similar to training data bias discussed later in this manuscript, but it only includes differences related to population characteristics, whereas training data bias is more comprehensive, including other types of differences such those in image acquisition that may not correlate with demographic differences or dataset shifts. Algorithms can be overtrained to one group, reducing their ability to be applied in a useful way to other groups, due to bias in data collection that are consequently reflected in model development and model evaluation. It may also foster a lack of confidence in the algorithm’s utility for some populations and discourage further development and resourcing. For example, more significant and serious outcomes from COVID-19 have been observed in some age ranges, races, and/or ethnicities than others.^22^^,^^23^ Some socioeconomic groups may not have sought care for COVID-19 as soon as some other groups, and this may have resulted in their illness being more advanced before imaging was conducted. Or some populations may not have had CT scanning as a part of their care (or be referred for CT later in the disease process compared to other groups), and thus their data may not be included when training models.^24^ Population bias can be measured through cross-population modeling.^22^^,^^23^ The results of cross-population modeling can help researchers identify if a data harmonization procedure is needed to account for systematic differences in the populations that are relevant to AI/ML modeling and testing.

### 3.7 Temporal Bias

Temporal bias is common and has far-reaching implications. It can arise from differences in populations and behaviors over time, from the collection of development/validation data that is not representative of future real-world clinical data, or from the correlation of reader performance and state of knowledge of the disease at the time of clinical validation. Temporal bias also occurs when out-of-date data are used to train an AI/ML model. Data used for training an AI system can be different from current data for many reasons. Clinical practice changes over time. Data acquisitions also evolve and improve over time. These are problematic because algorithms may not be generalizable over time due to changes in disease process, differences in individual patient trajectories, and advances in the state of clinical knowledge. It is important to ensure that datasets for model development and evaluation are relevant to current clinical practices. Clinical practice changes over time. Data acquisitions also evolve and improve over time. If an AI/ML system is trained on out-of-date data, the performance of the algorithm may degrade when applied to current data. This bias can be measured by assessing differences in algorithm performance between testing on historical and current datasets. For example, in the early days of the COVID-19 pandemic, effective treatments were limited or not available. Using imaging and other patient data from 2020 to develop AI/ML models intended to predict hospitalization and outcomes might not be accurate for predicting patient outcomes for current COVID-19 patients in 2022 and beyond. Also since a disease, such as COVID-19, is known to evolve over time, a dataset can include subjects with different variants of the disease or different vaccination statuses. Vaccinated subjects with COVID-19 could potentially have different disease features than unvaccinated subjects, including the period of their disease trajectory and outcome.

Clinicians, whether practicing clinical care or participating in reader studies, may have different skills and abilities in assessing the novel disease earlier in the course of the disease than those same clinicians (or others) later. This can potentially affect the reference standard for model development and model evaluation, which in turn can affect model performance at deployment. Temporal bias can additionally inflate observed associations and effect sizes. It is important to be aware of ways, in which data may have shifted over time. Temporal bias can be assessed through temporal validation (e.g., applying the model to a set of more recent cases after development using earlier cases),^25^ temporal validation plots,^26^ and intraclass correlation coefficients.^27^ Yuan et al.^28^ described several mitigation strategies for case-control study designs, including clear definition of baseline dates in cohort studies.

### 3.8 Sampling/Representation/Selection Bias

Sampling/representation/selection bias occurs when patient data used for training/tuning/testing an AI/ML model is not representative of the patient population to which the algorithm is intended to be applied. For example, if data collection is based on convenience and availability, without sufficiently considering the clinical task and patient population, this may result in sampling/representation/selection bias. Thus the collected biased dataset may lead algorithms astray in at least two ways: (a) shortcut over signal: because of incorrect sampling the algorithm may be prone to learning from confounding factors instead of the pathology of interest (e.g., an AI/ML model learns to identify breathing tubes rather than the COVID-19 opacities for which it was intended).^29^ (b) Data drift: the algorithm learns from the pathology of interest, but because of data drift, the performance on the intended population has systematic deviations from estimates on study samples. Such data collection bias results in performance estimates that are not generalizable to the intended patient population of the AI/ML model, resulting in questionable effectiveness and trustworthiness of AI/ML. Selection bias can arise, for example, when data selection correlates with disease severity or when a single institution serves as data source for a particular task. Data collection from multiple sources can help mitigate this bias, as can curation by matching key characteristics of the intended patient population.

### 3.9 Activity Bias

High-income regions are more likely to have the equipment, infrastructure, and personnel to enable data collection, research, and development of AI/ML models. Activity bias may occur when the collection of training data is biased toward regions that are rich in clinical activities involving use of certain modalities/equipment and rich in research activities involving archiving data and developing models.^30^ Caution must be taken when such models are deployed to regions that lack such activities. When models are deployed to such regions (e.g., low-income regions) that are not active in archiving patient data for AI/ML development, performance may be lower than expected. This bias can be measured by comparing metrics usually used in AI/ML performance assessment (such as area under the ROC curve, sensitivity, and specificity) on independent datasets from different regions (e.g., high-income regions that contributed training data versus low-income regions that did not), together with a comparison of patient characteristics.

## 4 Bias Category II: Data Preparation and Annotation

Data preparation and annotation are key elements of the supervised learning AI/ML chain. Annotators can be the source of substantial bias, e.g., content production bias and presentation bias listed in this section (Table 2) but also reference standard bias, institutional/systemic bias, historical bias, etc. (described in other sections) in their annotations.

**Table 2:** Overview of biases mainly associated with data preparation and annotation.

Bias source	Definition	Potential mitigation
Annotator bias	Occurs when human annotators, or human–computer assisted systems, apply subjective, selective, and/or biased labels in the annotation process	Inclusion of multiple annotators with different levels of experience; inclusive, diverse, multi-institutional annotator pools; intra- and interannotator comparisons
Content production bias	A form of behavioral bias that is expressed as lexical, syntactic, semantic, and structural differences in the content generated by users. These differences may impact the generalizability of research that utilizes user-generated content like annotations or patient-reported information	Seek diverse input from the appropriate populations (cultural, professional, geographic, etc.) based on the type of data collected to better understand how the content was (or will be) generated
Presentation bias	Results from the way in which images, AI/ML output, or other data are presented to the user or the annotator	Careful design of user interfaces and order of presentation for images and data. Quantitative comparison of different presentations schemes

### 4.1 Annotator Bias

Are AI/ML models learning the “truth” or modeling annotators? Every annotator has habits, experience, and elements of subjective judgment that impact data labeling and may lead to bias. Annotators will inevitably project such biases to their annotations. Prior rater experience, training, historic context, image preparation and presentation, annotation system user interface, and others, all contribute to a degree of subjectiveness in annotator judgment when presented with a case. This may lead to intra- and interannotator variability and bias. As such, the term “reference standard” is preferred over “ground truth;” in practice, nothing as absolute exists as the word truth would suggest. If the reference standard is tainted, noisy, inconsistent, or biased by annotator bias (through individual, historic, training, experience, content production-, presentation-, and reporting bias etc., see below) algorithms developed with such biased data are likely to reproduce, spread, and even increase existing bias, with lower accountability. Measures of intra- and interrater variability are one way of quantifying annotator bias at the entry point of model development. Synthetic or phantom images and image datasets may be used to probe the impact of annotator bias in algorithmic output. Best annotation practices should include multiple annotators, intra- and interannotator comparisons; annotator pools should be inclusive, diverse, multi-institutional, and—depending on the desired endpoint—include annotators with different levels of experience. Moreover, to mitigate bias, real-world testing of algorithmic performance should not only focus on testing against diverse independent datasets but also, when possible, involve testing against an independent pool of annotators. That is, to establish a reference standard for model performance testing, the use of annotators independent of those for the training data is helpful to avoid self-consistency bias. Annotator instructions and deployed tools should be carefully documented and preferably standardized. For spatial information [e.g., regions of interest (ROIs)], methods, such as simultaneous truth and performance level estimation and other similar methods, may be used as mitigation.^31^

### 4.2 Content Production Bias

Content production bias arises from systematic distortions in behavior across platforms, contexts, or different datasets that are expressed as lexical, syntactic, semantic, and structural differences in the content generated by users. Note that in this context a “user” is not an investigator but rather an individual who has generated data used in an AI/ML pipeline, e.g., an annotator or a patient. The use of language(s) varies across and within countries, populations, cultures, and professional domains. This variation includes influences from the type, level, and era of the users’ education, training, and experiences. Content production biases can affect the external validity (or generalizability) of research.^32^ Further, these biases raise additional concerns as they can affect several popular tasks, such as user classification, language identification, data characterization, and content filtering, and may also impact users’ exposure to a variety of information types (that is, these biases may influence users’ experience, or lack of experience, with particular types of data).^1^ For example, suppose a group of annotators with different professional backgrounds is asked to characterize a set of CT scans based on various acquisition factors with only minimal guidance. Some may refer to a characteristic such as dose modulation by its generic name, others may refer to it by the different trademarked names used by vendors, and some may not refer to it at all. Another example could be the annotation of findings observed in imaging exams, again with minimal guidance. Some may use the term “tumor” only for cancers, whereas others may use it more generically (benign as well as malignant). Factors like these could lead an investigator to misinterpret the generated annotation data. Thus there is a clear need for well-defined annotation guidance and the use of standardized lexicons when available. It is important to seek diverse input from the appropriate populations (cultural, professional, geographic, etc.) based on the type of data collected to better understand how the content was (or will be) generated. It is also helpful to make the guidance and lexicons used by annotators available to the investigators who will use the data.

### 4.3 Presentation Bias

AI systems interact with users in complex ways.^33^ Likewise, in data preparation or annotation generation for AI systems, there are complex interactions between the human and the computer. During data preparation and annotation, data may be presented to the human in a way that systematically affects their input or annotations compared to the true state of the data or patient. Likewise, during model evaluation in a reader study, data may be presented to the human in a way that may systematically alter their decision compared to how the device would be used in the clinic. These systematic differences will result in a bias. Presentation bias during the data preparation stage may result in biased annotations that limit the training and, therefore, the effectiveness of the AI/ML system and could also result in systematic differences between different subpopulations. Presentation bias during model evaluation may result in a difference between the measured and true (clinical) performance of the AI system. Moreover, it is important to note that the magnitude or impact of presentation bias may depend on the level of expertise of the human reader and on their subspecialty.

Consider a scenario for data collection to train an AI/ML system for detection and classification of COVID-affected regions of the lung. The developer has already developed a prescreening technique to detect ROIs and now wishes to collect training data to help design a classifier to classify these ROIs as positive or negative. In training data collection for this classifier, two ROIs are shown to an expert, who is tasked with selecting the ROI that is more likely to be diseased. The user interface for this data collection method is designed such that the ROI that received a higher score from the prescreening stage is always presented on the right-hand side of the screen. The human user is likely to realize this design peculiarity and may consciously or unconsciously rate the ROI on the right-hand side to be more likely to be diseased compared to a condition where the two ROIs are placed randomly on the right or the left. The AI method trained with this biased training set may not be able to reach the level of attainable performance. This type of bias may be hard to detect or measure during data preparation/annotation because the downstream effects may be difficult to pinpoint and compare. If a particular contributing factor for presentation bias is suspected during data preparation/annotation, one can design an experiment with/without the contributing factor to study the effect, using the same metrics that would be appropriate for the evaluation of the final AI/ML model. Experts in human–computer interfaces may be consulted to minimize this type of bias. For detecting or measuring this bias in model evaluation, differences in performance during the evaluation versus clinical use may be used. There may be several factors contributing to such differences, and presentation bias should be kept in mind as a contributing factor.

## 5 Bias Category III: Model Development

We identified six potential sources of bias mainly associated with the development of an AI/ML model (Table 3).

**Table 3:** Overview of biases mainly associated with model development.

Bias source	Definition	Potential mitigation
Inherited/error propagation bias	Occurs when machine learning models are used to generate inputs for other machine learning algorithms or trained incrementally	Under- or oversampling of training data used along the pipeline to mitigate any biases introduced earlier on and/or training with different random seeds
Reference standard bias	Occurs when there are inconsistent reference test methods, inconsistent procedures in which a given test is performed, inconsistent ways in which results are interpreted, or ignoring indeterminate findings	Use the highest possible level of reference standard that correlates with patient outcomes
Membership bias	Occurs when membership in particular groups present systemic differences that do not necessarily correspond with to the outcome of prediction being pursued in the target population	Optimization of model parameters and training size. Balancing the difficulty of the classification task relative to the difficulty of group membership recognition
Historical bias	Arises from systemic societal, institutional, and individual, engrained biases and impacts prioritization of problems to pursue	Construction of a more inclusive research community and consultation with social researchers and other subject matter experts
Training data bias	Occurs when there is a mismatch between the training set and the intended use	Adequate assessment of the diversity, or lack thereof, of one’s data
Cognitive bias	Arises when a system of belief, typically built upon data of limited validity and sets of heuristic, subjective assessments of physical quantities or outcomes, used to reduce the complexity of tasks produces systematic bias/errors in judgement of the underlying reality	Awareness of the bias introduced by the human operator is needed; develop clear knowledge of the applicability and limitations of the AI/ML model

### 5.1 Inherited/Error Propagation Bias

This type of bias occurs when machine learning models are used to generate inputs for other machine learning algorithms or trained incrementally, and bias is inherited by the subsequent algorithms in the pipeline. It is quite common that machine learning models are used to generate inputs for other machine learning algorithms. In clinical practice, a typical medical image analysis pipeline involves multiple sequential steps, such as image preprocessing, image registration, segmentation, and classification. AI/ML models are increasingly used for each of these steps, and the propagation of output from one step to the next can lead to an accumulation of errors, i.e., error propagation bias, that may affect performance for the clinical task at hand, such as lesion classification for malignancy or assessment of patient response to therapy, not only for human observers but also for downstream AI/ML models. Recent research has been done to investigate the mitigation of this bias and it has been demonstrated how uncertainty estimates can be used to improve downstream model performance. A general framework for propagating uncertainties across different classes of inference steps has been proposed for this purpose.^34^ For such a downstream improvement in performance to occur, each model in the chain needs to output its uncertainty estimates. Very few models used in the clinic provide uncertainty estimates. One should also note that models can be “confidently wrong,” in which case uncertainty estimates may be of little use (Sec. 7.4).

Propagation of errors is also possible when models are trained incrementally as in transfer learning; this time not because of errors/bias in AI/ML output, but due to the “freezing” of part of the model weights and architecture as it is trained and fine-tuned in consecutive steps using different datasets. Along this pipeline, potential biases in the datasets as well as any errors in the model weights may propagate into the final trained model. This approach is often used in progressing transfer learning or curriculum learning where the first step is often to train on a large set of natural images like ImageNet^35^^,^^36^ and then progressively fine-tuned on more relevant, usually smaller, medical image datasets. For example, when an AI/ML model was developed for the diagnosis of COVID-19 on chest radiographs, a model pretrained on ImageNet was used and then subsequently fine-tuned on the NIH Chest-Xray14^37^ and the RSNA pneumonia chest radiograph datasets^37^ before further fine-tuning on an in-house COVID-positive versus COVID-negative set of chest radiographs.^38^ Any potential biases introduced in any of these steps could have been propagated into the final trained model. Potential mitigation strategies include under- or oversampling of training data used along the incremental learning pipeline to mitigate any biases introduced earlier on.

### 5.2 Reference Standard Bias

A reference standard refers to the best available method for establishing the presence or absence of a condition of interest or extent of disease. When validating an AI/ML model, there needs to be a consistently high standard for the data used during assessment and it is essential to have a high-quality reference standard against which the new technology can be assessed.^39^ AI/ML models require unique considerations for the evaluation of potential optimal clinical utility, including retrospective or prospective validation in representative clinical settings, as well as establishing benchmarks against reference standards. When the reference standard comprises human expert annotations (see “annotator bias” above), the reference standard may not correspond to real-world clinical outcomes. When this happens, the AI/ML is developed and/or validated using a specific clinician or group’s assessments, not a generally applicable patient level outcome. But reference standard bias is broader than human annotator bias. It is a well-known problem that reference standards may be “fuzzy” or lacking; several reference standards may exist for a given condition, often with a “hierarchy” from high-level (more expensive, perhaps not widely available) to lower level (less expensive, more widely available but perhaps not as accurate). Thus problems with establishing a reference standard include inconsistent test methods, inconsistent procedures in which a given test is performed, inconsistent ways in which results are interpreted, or ignoring indeterminate findings.

When possible, AI/ML should be trained with the highest possible level of reference standard and, in testing, AI/ML outputs must likewise be compared to a rigorous standard that correlates with patient outcomes to get an accurate assessment of the safety, efficacy, and equity of the AI.

### 5.3 Membership Bias

Membership bias occurs when membership in particular groups present systematic differences that do not necessarily correspond with the outcome of the prediction of interest in the target population. When race or other specific demographic memberships are used as an explicit predictor or covariant in an AI/ML model, it could lead to bias if not applied properly. Patients of a given membership could collectively experience different factors that lead to their data having a systemic difference compared to another membership, but these factors may not be inherently related to health status and outcome alone. Certain members of a group could experience a systematic bias in the predictions made from a model that possesses membership bias, and this could impact decision-making made from those models. For example, if there is a correlation between race and access to health care, this could result in patients of one race membership having health data that is systemically different than the data of another race membership. This could result in models having a bias in terms of race that is more reflective of socioeconomic status, and it could also have a negative impact on the predictions made for test subjects of that race membership. Such bias may be amplified in AI/ML models’ predictions.^40^ Mitigation strategies for this bias include optimization of model parameters and training size^41^ and balancing the difficulty of the classification task relative to the difficulty of group membership recognition. If the classification task is more difficult than recognizing group membership, the AI/ML may learn to recognize group membership rather than perform the classification task of interest and bias is amplified. On the other hand, if the classification task is easier than recognizing group membership, bias is dampened in the early stages of training.

### 5.4 Historical Bias

Historical bias is a systemic bias, arising from societal, institutional, and individual, engrained biases, impacting prioritization of the relevant problems to pursue (model development), yet transversal to all bias categories (data collection, data preparation, model evaluation, and model deployment). Historical bias (as all systemic bias) is pervasive and insidious, often present in the “state of the art” of medical practice through legacy and discriminatory practices (e.g., so-called racial correction in pulmonary function tests, estimated glomerular filtration rate, and x-ray dose).^5^^,^^42^^,^^43^ Historical bias is also reflected in unequal access healthcare; for example, in COVID-19, and likely in other diseases, groups with less access to healthcare and or historical grievances related to healthcare, present a higher disease severity at first encounter with care. Historical bias also impacts the geographic heterogeneity of pollution exposure, housing quality, reliable access to high-quality food, and other factors that significantly impact health and outcomes. Historic bias is separated from temporal bias, as it is distinct from the natural evolution of biomedical knowledge, underlying technology, population changes, and medical education. It arises from historical and present systemic racism, gender stereotyping, and discrimination that sometimes clearly, often inconspicuously, is present in data collection, prioritizing of research, preparing data, and determining the reference standard and outcomes.

Exclusion of protected characteristics was sometimes proposed as a partial solution to historical bias; recent work has shown AI ability to infer withheld information (e.g., gender, age, and self-reported race),^44^^,^^45^ so this approach is not viable. Acknowledging and understanding the presence of historical and systemic biases and their impacts is the preferred mitigation tactic. The creation of a more inclusive research community will certainly contribute to a fairer prioritization of research and technologies to pursue. Similarly, collaboration with social researchers and other subject matter experts may contribute to minimize historical bias by understanding and correcting for how historical factors may shape data distributions for specific groups and by understanding and correcting for how a “tainted” reference standard and external factors may affect data and algorithmic performance.

### 5.5 Training Data Bias

Training data bias occurs in AI/ML when the data used to train the AI/ML does not represent the population for which it is intended, i.e., there is a mismatch between the training set and the intended use, and the populations of interest are not adequately represented.^46^ When training bias occurs, an AI/ML model may skew its output based on the prevalence of disease(s) in the training data, potentially over or underrepresenting the prevalence in the target population on which the AI/ML will be deployed. Data generation bias for AI/ML training can be seen as a subset of training data bias. In data generation bias, the emphasis is on how the images are generated, i.e., equipment, acquisition conditions, and sites. Training data bias includes these biases but also includes differences in populations, e.g., inadequately represented subpopulations. Note that training data bias is related to data shift since data shift will result in a different represented population distribution during deployment when compared to training. Training data bias may yield an AI/ML model that gives prejudiced results due to erroneous assumptions on the data, and the real-world AI/ML model’s performance may be lower than expected. A commonly used example is racial discrimination in facial recognition technology.^47^ In medical imaging, examples include AI/ML trained on single institution data, single acquisition data, or on populations of different races. Another relevant example is if only one person generates annotations to provide a reference standard for training (see annotator bias above). Training data bias can be detected/measured when different performance levels are obtained on different populations.

### 5.6 Cognitive Bias

Integrating the real world and making decisions is a burdensome task for the human brain and AI/ML alike; reverting to simpler approximations and heuristics may “automate” several decisions.^48^ Cognitive bias occurs when automated “simplifications” take over a decision process. For medical imaging, heuristics of representativity (“this case looks like the textbook case,” hence it must be), availability (“I remember one just like this”), and adjustment anchoring (“I have an idea that I will use as a starting point and adjust from there”) are relevant examples. Cognitive bias has the potential to bias reference standard labels (e.g., in directed annotation tasks), while also carrying the potential for over-reliance on (and/or dismissal of) algorithmic output. Cognitive bias potentially impacts multiple levels of imaging AI/ML, from setting a reference standard in supervised learning, impacting model development, to model deployment, especially in systems where AI/ML is assisting human experts in complex tasks. Well-designed experiments can account for and quantify cognitive bias; testing priming/anchoring, for example, including preselected sets of examples with deliberate different primers in an AI-assisted diagnosis task can provide a measure of cognitive bias.

## 6 Bias Category IV: Model Evaluation

Four potential sources of bias were identified, mainly associated with AI/ML model evaluation (Table 4).

**Table 4:** Overview of biases mainly associated with model evaluation.

Bias source	Definition	Potential mitigation
Evaluation bias	Arises through improper benchmark datasets, improper use of data or performance metrics	Use well-curated up to date benchmark datasets; proper data stratification and performance assessment
Detection bias	Refers to systematic differences between different groups in the detection rate or severity evaluation for a disease or condition	Use clinical knowledge and/or tightly controlled studies to uncover and quantify this type of bias. Then let the evaluators know about it so that they may use statistical techniques to appropriately compare the model performance for different groups
Amplification bias	Occurs when an AI/ ML algorithm learns to predict output/classes with a greater disparity than what is in the underlying ground truth	(i) Use appropriately balanced training sets or (ii) conduct influenced-directed feature removal
Statistical bias	Is the average difference between a quantity we estimate from data and the actual value of the quantity	Provide references or analytical expressions for the estimation method and a rationale for the choice of estimator. Sharing of data

### 6.1 Evaluation Bias

AI algorithms are often evaluated, and sometimes selected for deployment, based on evaluation datasets. Ideally, a public benchmark dataset is available and used for evaluation, but many algorithms are also evaluated on proprietary datasets, or a combination of benchmark and proprietary datasets. Although the problems of representativeness are similar whether one has a benchmark dataset or a proprietary dataset, a benchmark dataset allows for algorithm comparison among investigators at large. It is important to note that if you are using a benchmark dataset for the selection of a model among multiple candidates, you should consequently use an independent evaluation dataset to evaluate the performance of the selected model in an unbiased manner.

Evaluating a system in isolation without understanding and modeling its real-world use may create unrealistic notions of its benefits and consequences. Thus the performance of the AI algorithm on the evaluation dataset may or may not be a good indicator of their performance on clinical tasks and for all subpopulations, depending on how the evaluation dataset was curated and whether the evaluation dataset was used for model selection. Evaluation bias can occur when the evaluation dataset is not representative of the use population or if the metrics used for the task are not representative of performance in the use task.^49^ Algorithms selected based on a nonrepresentative dataset may to lead to models that “perform well only on the subset of the data represented by the evaluation dataset.” As noted in Ref. 49, historical, representation, or measurement biases can be particularly problematic if they exist in the data. Algorithms are typically selected based on aggregate measures on the entire dataset and performance on subgroups is often not considered when evaluating benchmarks. For example, evaluation datasets for COVID-19 were established early in the pandemic and were not necessarily very diverse. There have been numerous variants since the original with potentially different characteristics on chest radiographs, and vaccinations have modified the associations between outcomes and risk factors. Models selected using evaluation datasets from early in the pandemic may not be optimal for later variants or for all subgroups.

Another cause of evaluation bias is duplication of data in the training, validation and test sets due to improper stratification. For instance, if stratification is not performed on the patient level but on the imaging study, or even on the level of an individual image or image patch, then not all information of a patient is kept together. All information pertaining to a given patient needs to end up either all in the training or all in the validation or all in the test set, respectively, or else performance will be substantially overestimated.

Along similar lines, evaluation bias may also occur based on the choice of performance metrics/figures of merit, e.g., when there are systematic differences between metrics in model development and deployment.

### 6.2 Detection Bias

Detection bias refers to systematic differences between different groups in the detection rate or severity evaluation for a disease or condition.^50^ Detection bias typically occurs because of differences between groups in how the disease is detected or evaluated. For example, if a very accurate detection method is very costly and therefore is not covered by low-cost insurance, this may result in a difference in disease detection rate between more and less affluent social groups. Detection bias may mask existing biases or may falsely lead investigators to conclude that there is bias. For example, prostate size is larger among overweight and obese men as compared to normal weight men, and as a result, the detection rate in biopsies for prostate cancer is lower in overweight and obese men.^51^ If the difference in the detection rate between normal weight and obese patients is not considered in epidemiologic studies, one may underestimate the association between obesity and overall prostate cancer risk, resulting in a biased estimate of cancer risk for obese patients. Another example is that in low-resource areas, COVID-19 tests may not be adequately calibrated or controlled such that the false-negative rate at these clinics may be higher than the average, and only very sick patients are diagnosed. An unbiased AI/ML model for the detection of COVID-19 on medical images may appear to have a sensitivity that is biased in favor of patients from low resource clinics because the detection rate for these patients is low, and only sicker patients whose images are easier for COVID-19 detection are included as actual positives. Detection bias can be assessed as the difference in disease detection rate or disease severity between different groups using studies, as tightly controlled as possible, that either consider/minimize some of the known factors affecting the bias in detection rate, and/or using technologies, approaches, and methods that have different (and ideally less) bias.

### 6.3 Amplification Bias

Amplification bias refers to the occurrences of erroneous output from AI/ML that amplifies already existing biases present in the training data (see training data bias).^52^ When a model amplifies bias, it makes certain predictions at a different rate for a subgroup than expected based on training data prevalence. Amplification bias occurs when an AI/ML model learns to predict output/classes with a greater disparity than what is in the underlying ground truth (e.g., class imbalance in the training set or training using biased features). This may occur, for example, if certain computer-extracted features, which are very specific to a subpopulation, dominate the predictive modeling. Amplification bias can lead to under- or overdiagnosis diagnosis in subpopulations, e.g., in the diagnosis of chest radiographs in underserved populations.^53^ It can be measured by evaluating the trained AI/ML model on different subpopulations or by training it with data with different prevalence. Mitigation of amplification bias can occur (i) using appropriately balanced training sets (avoiding training data bias) or (ii) conducting influenced-directed feature removal.^52^ Relevant to the latter, one can attempt to mitigate amplification due to features by enforcing parity in features across the classes, i.e., ordering features by their influence on the AI/ML’s output and remove features from the dominant class until parity is reached.^40^

### 6.4 Statistical Bias

Statistical bias is a broad term referring to a systematic tendency for there to be a difference between the true value of a parameter being estimated and the expected value of the parameter’s estimates. This systematic tendency can be either for the estimates or measurements to be above or below their true values. Statistical bias is often understood to be the result of an incorrect analytic estimator, e.g., the well-known difference between the sample variance and the population variance.^54^ However, there are many additional, often subtle and complex, sources of statistical bias that might enter into the development and evaluation of an AI/ML model. Statistical biases arise from systematic as opposed to random error and can occur in the absence of prejudice, partiality, or discriminatory intent. Statistical bias occurs when estimates of algorithm performance are systematically impacted by errors in data collection or analysis owing to incorrect statistical models. Practitioners may use a statistically biased estimator for practical reasons (perhaps an unbiased estimate of a parameter is not tractable). In other cases, practitioners may choose to use a biased estimation method because it has other desirable characteristics (nonnegative estimates of a known-to-be nonnegative parameter, or desirable bias-variance trade-off characteristics). Statistical bias can result in over- or underestimates of an AI/ML model’s performance, leading to algorithms being believed to be more effective than they are in some cases and not appreciated for the value they might provide in others. For example, performance levels of an AI/ML model in medical imaging are often estimated on units that are not independent, e.g., per lesion, per region of interest, and per lung lobe, as the ones from the same patient are likely to be correlated. Variance estimates are often biased if such correlations are not accounted for, thereby leading to incorrect (often overly optimistic) inference.^55^ The potential for bias in an estimation procedure can be known through simulation studies and assessed, e.g., through the difference between the mean of the parameter’s estimates and the true mean.

## 7 Bias Category V: Model Deployment

Model deployment is the final step in the medical imaging AI/ML pipeline. After all the work from data collection to thorough model evaluation, it is time to prospectively evaluate performance in the real world and perhaps even clinical practice. Seven potential sources of bias mainly associated with model deployment were identified (Table 5).

**Table 5:** Overview of biases mainly associated with model deployment.

Bias source	Definition	Potential mitigation
Deployment bias	Arises when there is a mismatch between the intended use of a system or algorithm and the way it is used in practice. This misuse may cause harmful decisions or consequences.	Systematically monitor and continually evaluate the model’s usage; consider a formal assessment (like a clinical trial or observer study) to evaluate usage outcomes
Concept drift/emergent bias	Occurs when the performance of machine learning models estimated in the laboratory setting degrades over time in the real world when the image acquisition equipment, clinical conditions, and patient population characteristics change	Retraining and reassessment with more recent data, which can be demanding in data collection
Behavioral bias	Arises through systematic distortions in user behavior across platforms or contexts, or across users represented in different datasets	Education and information, as well as directly addressing misinformation. Increasing access to healthcare and promoting a just, equitable, diverse, and inclusive healthcare experience for all
Uncertainty bias/epistemic uncertainty	Is the influence of both reducible (epistemic) and irreducible (aleatoric) uncertainty on decision making drawn from AI/ML models	Consider retraining a classifier or network with different or additional data.
Funding/publication bias	Arises through selective reporting of results	Transparency in reporting, publicly available data, open-source code
Automation complacency/loss of situational awareness bias	Caused by over-reliance on automation	Emphasize human accountability in machine–human interaction; awareness of the limits of applicability of algorithms
User interaction bias	Can occur when users interact with data and algorithmic outputs based on their inherent biases or a biased user-interface, impacting end user choices and decisions	Thorough design and testing at the level of the user interface

### 7.1 Deployment Bias

Deployment bias arises when there is a mismatch between the intended use of a system or algorithm and the way it is used in practice. For example, when a system not intended as a decision aid (e.g., a quantitative imaging tool or a segmentation tool) is used as a decision aid by human operators or interpreters, this practice will result in deployment bias. It is also referred to as the “framing trap.”^49^ Most systems will inevitably be ultimately deployed within complicated institutional structures and utilized by human decision-makers. The human intermediary may act on predictions in ways that are not included in the original intended use of the system; these differences may lead to misinterpretation or misuse of the output of the system. Misinterpretation or misuse of a system by the ultimate users, via phenomena like confirmation bias or automation complacency bias (see below), may lead to harmful decisions.^56^ Improper use or interpretation by end users can take many different forms, and it may be intentional or unintentional. For example, suppose a system has been developed that analyzes images from brain MRI scans and provides numeric assessments of various features. These features are known to have clinical utility and the system was trained, validated, and tested in the laboratory on a large database of images acquired from a variety of 1.5T MRI units. The system has now been deployed in a clinical setting. A simple technical misuse could be a radiologist utilizing the system to analyze images from both 1.5T and 3T MRI units. The system was only trained and validated with images from 1.5T units, and it is not known if it will maintain its performance with images from 3T units. Similar misuse might include utilizing the system to analyze images from different anatomy (e.g., abdominal or breast images instead of the brain) or even a different modality (e.g., brain CT images instead of brain MR images). In each case, this misuse could be unintentional (the radiologist may not be aware of the intended use of the system or of its documented limitations) or intentional (the radiologist is aware of the system’s intended use but has explicitly decided to proceed anyway, analogous to “off label” use). Misuse could also manifest as an improper interpretation or utilization of the system’s output. The use of the system’s output as a proxy for predictions or courses of action that have not been modeled (again, analogous to “off label” use) is problematic as the system’s performance in this context is unknown. To prevent this bias, AI/ML developers should consult with relevant stakeholders (radiologists or other physicians, medical physicists, department administrators, etc.) to understand how the model or system may be used when deployed. Technical safeguards should be in place to ensure only appropriate data is imported and analyzed by the system when possible. The AI/ML system should be designed so that the output of the system is human-interpretable. Similarly, user interfaces should be designed to help users understand the limitations of the system and effectively interpret system output. Finally, it is critical to monitor the deployment of the algorithm to understand its use in practice.^57^

### 7.2 Concept Drift/Emergent Bias

Concept drift occurs when the relationship between the input (e.g., images and clinical features) and the output (e.g., diagnosis and prognosis) changes over time due to data drifts, such as changes in image acquisition devices/protocols, disease prevalence, changes in the gold-standard technology, and virus mutation. For example, the COVID-19 pandemic is evolving over time, probably more rapidly and drastically than other diseases such as cancer. It has been shown that AI/ML models using blood tests to predict COVID-19 diagnosis are affected by this.^58^ Concept drift or emergent bias is usually measured by monitoring model performance metrics (e.g., area under the ROC curve, sensitivity, and specificity) over time.

### 7.3 Behavioral Bias

Behavioral bias refers to systematic distortions in user behavior across platforms or contexts, or across users represented in different datasets.^32^ Prior experience, stigmatization, exposure to misinformation, limited access to healthcare, and historic context all conjure to produce subjectiveness and irrationally driven behavior of users in relation to healthcare, medical imaging, data sharing, etc., with transversal impact across the development process. Behavior bias has the potential to impact all levels of imaging AI/ML from data collection to model deployment. It has the potential to skew cohorts, produce missing information, and increase the uncertainty of outcomes, while also carrying the potential for dismissal of algorithmic-assisted medical advice. For example, in the COVID-19 pandemic behavior bias is introduced through self-selection (inclusion or exclusion, impacting availability of data), missing or misleading information (vaccine status, co-morbidities, prior COVID-19, smoking status, etc.), and increasing uncertainty in outcome measures (through lack of follow-up, disregard, or inability to pursue prescribed care, etc.). Data on self-reported vaccination may be impacted by behavior bias, as well as prior COVID-19 exposures through self-exclusion. Smoking status is another classical example. Self-selection through inclusion and/or exclusion is also a potentially significant source of bias in existing datasets. More importantly, for communities historically discriminated against interactions with healthcare, severity of COVID-19 at the time of the first encounter with healthcare may be biased toward higher severity, and less severe cases may be underrepresented. Specific components of behavior bias can be assessed through thoughtfully crafted sociologic experiments indirectly,^59^ through severity of disease at time of presentation, and via differences observed between distributions (e.g., census versus data collected or seropositivity versus self-reported COVID-19, vaccine status). Targeted community outreach, when behavior bias is identified in specific groups, may be used as a mitigating factor toward behavioral bias, e.g., relating to self-exclusion. Acknowledging the presence of behavior bias allows for strategies that are common to other mitigation strategies, such as cohort balancing, standardized reporting, and documentation on the limitations of developed algorithms, including the representativity of the training dataset and real-world test of claims against independent, representative, dataset(s) (sequestered data).

### 7.4 Uncertainty Bias/Epistemic Uncertainty

Uncertainty bias is the influence of both reducible (epistemic) and irreducible (aleatoric) uncertainty on decision-making drawn from AI/ML models. Characterizing and estimating/measuring uncertainty is essential to robust AI/ML model evaluation and transparent reporting. However, human observer decisions made based on AI/ML model output and reported uncertainty thereof can be overly swayed by the uncertainty in model output. For example, it is known that AI/ML models can be “confidently wrong,” i.e., they can produce incorrect outputs with high confidence.^60^ If this occurs, humans may place more value on a prediction that has high certainty, even if it is incorrect, than on one that has low certainty but is correct. This could influence how an AI/ML model is used for subsequent decision-making, either for further algorithmic work or clinical implementation.^61^ Some research has been done to develop “reliability maps” (which are not actually spatial maps but rather a measure of “reliability” across the posterior probability output).^62^ It is a measure of uncertainty using variance and a “calibration map,” based upon a measure of prevalence.

### 7.5 Funding/Publication Bias

Researchers tend to accentuate the positive—whether motivated by expectations of a funding agency, an employer, colleagues, or for any other reason.^63^ By picking and choosing what to publish/report on, a bias is introduced that can lead to bad science. Funding bias refers to selectively reporting results to support or satisfy the expectations of the funding agency or financial supporter of a research study. Publication bias, similarly, and more generally, refers to the selective publication of results based on the outcomes found. Different subtypes of biases fall under publication bias: (i) outcome reporting bias refers to publishing only the results in a study that appear positive, while failing to report those that appear negative (cherry-picking of results), (ii) spin refers to using language to make negative results appear positive, and (iii) citation bias refers to the fact that articles mentioning positive results tend to be cited more frequently than those reporting negative results. For good science, it is important to know when something does not work or how many failed attempts resulted in a success (false-discovery rate, p-hacking), which is especially important in medical imaging AI/ML studies where often many hypotheses are investigated simultaneously. Mitigation strategies include accurate and transparent publication of all methods, protocols, and results, regardless of findings, and development of—and adherence to—rigorous standards for publication.

### 7.6 Automation Complacency/Loss of Situational Awareness Bias

Automation complacency occurs when humans over-rely on automation, or automation leads to humans being unaware of their situation such that, when control of a system is given back to them in a situation where humans and machines cooperate, the skill of humans are attenuated, and they are unprepared to assume their duties. This can include a loss of awareness over what automation is and is not taking care of and its limitations.^64^ Attention plays a major role in this bias; it is worsened when multiple-task overload (emergency), complexity, and/or fatigue is present in the human expert. Human attention being finite, overreliance on automated or semiautomated decision support, particularly in repetitive tasks, under pressure, or in complex situations, may lead to errors both of omission and commission.^65^ Typical examples are linked to aviation, where automation bias and automation complacency have been found to be the direct cause of accidents. In our context, automation bias, automation complacency, and loss of situational awareness bias is relevant at any interface where algorithmic output and human interaction is present, namely in decision support and automation. It is more relevant in model deployment, potentially also in algorithmic-assisted annotation. It has the potential to lead to erroneous decisions and relevant omissions, directly impacting individual patient health. More generally, as with many other biases, consequences of this bias may undermine public trust in algorithmic decision-making support systems (at the deployment level) and may bias new and existing datasets when used in the process of annotations and establishing a reference standard for training. It can be quantified with controlled experiments where ground truth and confounders are well established or characterized. These studies also allow for comparison of mitigation measures. Awareness of the bias by the user is a key, as it is training and knowledge specific to the algorithmic process, its known limitations, and limits of applicability. Other mitigation strategies include emphasizing human accountability in algorithmic assisted processes, as well as communication and design changes with algorithmic output labeled as “information” (as opposed to “recommendation” or “decision,” for example) and user interfaces highlighting “raw data” rather than the algorithmic output, as well as adding confidence intervals to algorithmic outputs. User experience is also important, as well as individual differences linked to confidence and trust.

### 7.7 User Interaction Bias

User interaction bias may arise when a user imposes self-selected biases and behavior during interaction with data, output, results, etc. It also arises or can be driven by the interface between the user and the automated system (related to presentation bias, such as inadequate graphic user interface, systematic bias in presentation, and ranking of the information presented).^1^^,^^32^^,^^66^ In contexts when the user is the “end user” with ability to make unconstrained choices, user interaction bias is also referred to as “consumer bias.” Humans impose their own set of biases on interaction with data and outputs. This can lead to a self-reinforcing loop: bias being reproduced and augmented by algorithms, which in turn will reinforce existing bias. Typical examples are linked to presentation bias and ranking bias (what is shown first “must” be more important), both of which are self-reinforcing. User interaction bias has the potential to undermine trust in algorithmic decision at the deployment level, and more perversely, it may produce actions and/or new data tainted with bias, which will in turn reinforce existing bias while masking responsibility and the origin of bias through algorithmic complexity. For example, in a graphical user interface where COVID-19 positive/negative outcomes of an AI/ML model are prominently displayed, users may miss other relevant information contained in the image, such as the presence of other markers of respiratory or cardiorespiratory disease. In a different example gender, race, age, scanner type, local circumstances, etc., inferred or explicitly displayed when analyzing medical images may, combined with the user’s pre-existing bias, amplify the human bias. User interaction bias is hard to measure and awareness of this potential bias by the user is a key. In the consumer space, clear communication and education, including the use of role models and trusted channels, have been shown to mitigate user interaction bias. Components of user interaction bias can be measured and then mitigated through well-crafted controlled experiments, where the information displayed, ranks, presentation, and graphical user interfaces should be deliberately manipulated to test the impact on the end user.

## 8 Discussion

With the increased popularity of modern AI tools in medical imaging and the public availability of many (often pretrained) AI/ML models for (mostly) natural images, the threshold for developing a medical imaging AI/ML model has been lowered substantially. This is advantageous since it allows for relatively easy translation and adaptation of techniques developed in other research domains to medical imaging analysis. However, a lack of domain knowledge may result in nonexistent or faulty early data discovery analysis, inappropriate study design, and incorrect performance evaluation, which likely lead to a multitude of biases and lack of generalizability of the end result.

Federated learning is sometimes thought to solve many of the potential biases described in this paper. It has been proposed as a class of techniques used to build more robust models by allowing models to learn from multi-institutional data without the need for data sharing.^67^^–^^69^ The idea is that models built from a diverse dataset representing many populations with data acquired on different systems may be less biased than models built from a single site, may be able to handle differences in labeling practices,^70^^,^^71^ and generate models that are more consensus-based compared to models trained on single institution data. However, federated learning may have similar concerns to centrally hosted repositories in terms of challenges with data heterogeneity. Many of the biases discussed here can occur even in federated learning scenarios.^69^ Deep learning models can still be brittle and may not extrapolate or generalize well, i.e., they may still not work well on data that are not represented in the training dataset. Moreover, depending on the technical approach to federated learning, models may be “biased” toward data from sites that contributed more data.^71^ Federated learning may also introduce bias because, among other factors, (1) the technical requirements to set up federated learning may prevent sites without sufficient IT support from participating in the activity, (2) lack of standardization may exist in data acquisition protocols and labeling practices between sites may vary between sites, and (3) noisy or discordant labels between sites may reduce the overall performance of the models and/or introduce bias. Thus although federated learning has the potential to reduce some of the biases related to data heterogeneity, it is not necessarily a solution to many of the concerns raised here.

The work presented in this paper is complementary to existing efforts, such as those by National Institute of Standards and Technology (NIST),^2^ intended to provide a standard for identifying and managing bias in AI/ML in general. The NIST bias list in the glossary of Ref. 2 is extensive and comprehensive, but at times not directly applicable or translatable to medical imaging. Thus we note that the list developed by NIST includes additional sources of biases that may not apply to medical imaging and, in some cases, we rephrased their language for a medical imaging audience. Specific to medical imaging research, a few notable efforts have been undertaken within the last few years to promote better quality research studies and publications within the medical imaging AI/ML research domain.^6^^–^^8^^,^^39^^,^^72^^–^^78^ These publications provide recommendations, frameworks, and/or checklists to define a minimum desired quality standard when it comes to medical imaging research study data, methods (including statistics), and transparency in reporting. One such effort is the idea of “dataset nutrition label,”^79^ where curation, including quality, representativity, and qualitative factors can be provided as a fact sheet for AI developers to understand the dataset, acceptable uses, and limitations, at the point of problem definition.

Our work is synergistic with these prior efforts by (i) providing an overview of biases tailored to medical image analysis AI/ML, (ii) identifying their occurrence in one or more of the five steps along the AI/ML pipeline from data collection to clinical translation, and (iii) providing detailed bias descriptions including similarities, nuanced differences, and recommendations for mitigation. Some sources of bias might only occur in the process at a single stage, while others have the potential to arise in almost every stage (Figs. 1 and 2). Membership bias is a notable example of this; members of a certain group might be underrepresented in the data collection process, might be more subject to annotation or truthing errors, might be more likely to be subconsciously subjected to bias in the selection of features or other aspects of model development, and might experience bias in the way a model is evaluated or deployed. Therefore, membership bias is a particularly important area for bias awareness as it can be a multiplicative source of bias in AI/ML models designed for medical image analysis. Membership bias is also an example of our comprehensive approach. Other authors might have chosen to merge certain sources of bias into a single category to yield a shortened list. We chose to be comprehensive and distinguish between subtypes to ensure that bias categories familiar to a given reader would be found within our presentation and nuances could be explained. Thus we provided explicit listings and definitions for exclusion bias and institution/systemic bias while these could be argued to be membership biases. We believe that it is useful to specifically define these sources of potential bias so that they are more likely to be accounted for and mitigated.

In summary, we have provided a comprehensive list of potential biases that can arise during one or more of the steps of the medical imaging AI/ML pipeline, from data collection to clinical model deployment. We also discussed mitigation strategies. Fairness and equity issues may arise for all these biases when there are systematic correlations between the data biases and any feature of equity concern e.g., demographics, socio-economic factors, geography. The work presented here is being translated into an interactive online tool available to researchers at large^80^ and fairness and equity will be one of the foci of the future work.
